# Regulatory safety evaluation of nanomedical products: key issues to refine

**DOI:** 10.1007/s13346-022-01208-4

**Published:** 2022-07-30

**Authors:** Wim H. De Jong, Robert E. Geertsma, Gerrit Borchard

**Affiliations:** 1 Formerly (retired) National Institute for Public Health and the Environment (RIVM), Bilthoven, The Netherlands; 2 National Institute for Public Health and the Environment (RIVM), Bilthoven, The Netherlands; 3 University of Geneva, Geneva, Switzerland

**Keywords:** Nanomedicines, Safety evaluation, Regulations, Immune system

## Abstract

Nanotechnologies enable great opportunities for the development and use of innovative (nano)medicine*s*. As is common for scientific and technical developments, recognized safety evaluation methods for regulatory purposes are lagging behind. The specific properties responsible for the desired functioning also hamper the safety evaluation of such products. Pharmacokinetics determination of the active pharmaceutical ingredient as well as the nanomaterial component is crucial. Due to their particulate nature, nanomedicines, similar to all nanomaterials, are primarily removed from the circulation by phagocytizing cells that are part of the immune system. Therefore, the immune system can be potentially a specific target for adverse effects of nanomedicines, and thus needs special attention during the safety evaluation. This DDTR special issue on the results of the REFINE project on a regulatory science framework for nanomedical products presents a highly valuable body of knowledge needed to address regulatory challenges and gaps in currently available testing methods for the safety evaluation of nanomedicines.

## Introduction

Nanotechnologies have developed very rapidly over the last decades, enabling important improvements in many types of products including medicinal products and medical devices (De Jong and Borm [1]; Soares et al. [2]; Geertsma et al. [3]). The possibilities for preparation of medicinal products applying nanotechnologies appear to be almost endless. Both synthetic materials like metal and polymer constructs and materials from a more biological origin such as lipid and protein constructs can be used in countless variations. In addition to the well-known COVID-19 vaccines based on mRNA in a lipid nanoparticle formulation, examples of already successful nanotechnology uses are gold nanoparticles for imaging, local hyperthermia, and drug delivery (Sharifi et al. [4]; Jahangirian et al. [5]; Park et al. [6]), liposome formulations for various highly toxic cytostatic drugs like doxorubicin and Cis-platin (Canão et al. [7]; D’Angelo et al. [8]; Park et al. [6]), albumin protein complexes for paclitaxel like abraxane (Park et al. [6]), and iron oxide for the treatment of anemia and for hyperthermic therapy (Jahangirian et al. [5]; Park et al. [6]). These various clinical applications show the success of the use of nanotechnology in medicinal products.

However, as always when golden opportunities present themselves, potential less favorable issues related to them need to be considered carefully as well. An important concern related to the use of nanotechnologies or nanomaterials in products has been how to demonstrate safety or determine an acceptable risk for such products. Already in 2009, the Scientific Committee on Emerging and Newly Identified Health Risks (SCENIHR) published an opinion on the risks of nanotechnology products (SCENIHR [9]). The most prominent statement was that nanomaterials should be seen as “normal” substances in that some of them are toxic and others are not. This statement presented a problem for risk assessors: how to conduct an acceptable and proper risk assessment for products utilizing nanomaterials? Nanomaterials are used for their specific characteristics that may differ considerably from their larger sized or soluble counterparts, conferring advantageous properties to the final product. The question will always be whether a change in physicochemical properties of such compounds will also change their toxicological properties. Therefore, it is assumed that a risk assessment of nanomaterials needs to be performed on a case-by-case basis.

Although this appears to be a straightforward approach, in practice it is not. For example, when can we safely assume nanomaterials of different sizes to behave in a similar way, i.e., to which extent does size distribution of nanomaterials play a role? And can we consider nanomaterials of the same size but of different chemical compositions to behave similarly when they both consist of solid insoluble particulate materials? Because of an almost infinitive number of nanomaterials that can be synthesized, such questions have initiated several research lines dedicated to read-across and grouping approaches for the risk assessment of nanomaterials (Oomen et al. [10]; Lamon et al. [11]; Mech at al. [12]; Braakhuis et al. [13]).

The particulate nature of nanomaterials has also opened new challenges regarding the evaluation of their interaction with biological systems they are exposed to. Especially for solid nanostructures this has resulted in the new field of nanotoxicology. Problems have been encountered regarding dosing in toxicological assay systems, characterization of the products including in-process controls and their specifications, the identification of critical quality attributes (CQAs), and the reproducibility of manufacturing processes: to which extent is the nanomaterial produced by one manufacturer the same as the one produced by another manufacturer? For nanomaterials, in general, and for nanomaterials used as or in nanomedical products these problems are similar. So far, safety evaluation of substances is based on general principles of toxicology. Bearing in mind the specific and particulate properties of nanomaterials, it is necessary to look beyond these classical toxicological paradigms. Especially their interaction with the immune system and resulting quick removal from the blood circulation by tissue phagocytic cells must be included in a more specific evaluation of nanostructures and their pharmacokinetic and toxicological profiles.

## What is a nanomaterial/nanomedicine?

The definition of a nanomaterial has been the subject of debate for many years now. For regulatory purposes a definition is desirable if specific provisions are considered necessary for products with specific properties. However, it is not clear which elements should be included in a definition to capture such properties. SCENIHR indicated that only the size of nanomaterials can be considered a unique property (SCENIHR [14]). Although there is no scientific rationale for this, a size between 1 and 100 nm has been selected in the EU recommendation for a definition in 2011 (EC [15]) and also in the international standard ISO TS/8004-1 (ISO [16]). For cosmetics (Cosmetics Directive 2009 and recast of 2016), novel foods, and for medical devices, the EU recommendation was largely followed in the regulatory frameworks (EU [17–20]). In June 2022, an updated version of the definition was published, replacing the 2011 version (EU [21]). The slight changes introduced will not significantly affect the scope of identified nanomaterials. For nanomedicines the EMA prefers to use a description rather than a definition, in which nanomedicines are referred to as tiny structures less than 1000 nm across, which are designed to have specific properties (https://www.ema.europa.eu/en/glossary/nanotechnology). A similar approach is followed by the FDA in their recently updated FDA guidance on drug products containing nanomaterials that states that structures up to 1000 nm may need special considerations even when their size is outside the nanoscale of 1–100 nm (FDA [22]). When we consider the particulate aspect of a nanomaterial/nanomedicine, we also need to look at the chemical substances used for their preparation. We can distinguish various types of nanomaterials: for example, those being a solid piece of matter or polymeric formulation that may be soluble, partly soluble, or insoluble, and other nanomaterials with a more biological origin already in use for decades in nanomedicines for drug delivery systems such as liposomes and lipid and protein complexes (Sercombe et al. [23]; Park et al. [6]). It can be easily recognized that the interaction with cells is quite different for a nanomaterial like a liposome, consisting of an outer wall of phospholipids or a protein complex than for solid Au, Ag, or TiO_2_ nanomaterials (Behzadi et al. [24]; Guo et al. [25]).

## What sets nanomedicines apart from other medicines?

The most striking characteristic of a nanomedicine is its particulate character, independent whether the formulation is of synthetic material or biological origin. This results in a biological behavior that is quite different from a soluble medicinal product. For intravenously administered nanomedicines, the toxico- and pharmacokinetic profiles are governed by the particulate nature of the construct. The blood circulation time and tissue distribution are not dependent on a concentration gradient like it is the case for soluble substances and medicines, but on the recognition by the immune system and the (active) removal of the nanomedicine from the blood (De Jong et al. [26]; Lankveld et al. [27]; Geraets et al. [28]). In addition, release of the active pharmaceutical ingredient (API) from the nanomedicine and partitioning into the blood during systemic circulation should be considered in relation to the drug carrier composition (Kovshova et al. [29]). Phagocytosis results in a relatively high concentration of the nanomedicine in the liver, spleen, and lung, organs rich in phagocytic cells that actively remove the particulate nanomaterial/nanomedicine from the blood. The active removal can be delayed by using a hydrophilic coating on the surface of the nanomedicine such as polyethyleneglycol (PEG) resulting in a prolonged systemic circulation time (Lankveld et al. [30]). Another possibility to direct tissue distribution of the nanomedicine is to add targeting ligands on their surface to promote, for example, specific uptake by a diseased (cancer) site, resulting in the local migration and extravasation as has been employed for doxorubicin (Serombe et al. [23]). Distribution of nanomedicines such as vaccines does of course differ upon subcutaneous and intramuscular injection with the potential formation of local depots from which the vaccine is removed by lymphatic drainage toward the lymph nodes (McCright et al. [31]).

## The role of macrophages

Macrophages are a part of the innate immune system. They play a central role in the immune response to obtain immunity by functioning as antigen presenting cells (APC) for further antigen processing within the immune system. They are among the first cells, after neutrophilic leukocytes, to respond to an external challenge of the body. When we consider the particulate nature of nanomedicines that end up to a large extent in the phagocytizing macrophages, this offers opportunities for specific directed targeting to deplete macrophages by liposome-encapsulated clodronate for therapeutic purposes (Ravichandran et al. [32]) or activate macrophages as antigen presenting cells for nanoformulated messenger RNA vaccines (Kiaie et al. [33]). However, the macrophage homing can have consequences with regard to possible adverse effects of the nanomedicine like chronic activation of macrophages in COVID vaccines resulting in immune dysregulation (Ravichandran et al. [32]). Via this specific exposure route, the immune system itself can become a direct target in terms of adverse effects or toxicity induced by nanomedicines. In addition to the immune activation, there may also be a toxic effect of the nanoconstruct itself or the encapsulated active API present in the drug carrier. So, the immune system can be targeted for specific therapeutic purposes but needs to be carefully watched for immune-related adverse effects as well.

## Safety evaluation of nanomedicines

For any product, the safety evaluation starts with the determination of the possibilities for exposure. For nanomedicines, this is the relatively simple part as the exposure will be determined by the therapeutic doses administered to a patient. Following administration, the toxico- and pharmacokinetic profiles are critical. Where does the nanomedicine go, what happens to the API—will it remain associated with the nanomaterial drug carrier? Before clinical trials can be started, an extensive preclinical safety evaluation needs to be performed. For nanomedicines, instead of traditional experimental animal models, simulation using in silico models is expected to become increasingly important to provide answers to these questions. Important resources for information on assays for the safety evaluation of nanomedicines are the NCI Nanotechnology Characterization Laboratory in the USA (Protocols - Nanotechnology Characterization Lab - NCI (cancer.gov)) and the European Nanomedicine Characterization Laboratory (Assay Cascade (euncl.org)). Both websites provide a large number of standard operating procedures for nanomedicine testing.

For nanomedicines, a prominent role for the immune system has been observed, as shown by a recent review of Halamoda-Kenzaoui and Bremer-Hoffman [34]. Indeed, this seems obvious as the immune system has a major role to remove foreign agents from the body. A comprehensive overview on immunotoxicity and methods for immunotoxicity testing of nanomaterials was published by WHO [35]. A dedicated strategy for the immunotoxicity testing of nanomedicines was recently proposed by Giannakou et al. [36].

The immune system is quite suited for the evaluation of various immune functions by in vitro assays. Immune cells can be harvested from various sources, including the blood of treated patients. This opens the possibility to follow an effect on at least parts of the immune system. Several in vitro assays are available that can be applied to evaluate nanomaterials/nanomedicines for their effect on macrophage and other immune functions (Vandebriel et al. [37, 38]). One specific issue is the presence of endotoxin as especially for immunotoxicity testing the presence of endotoxin can have a dramatic effect on the test outcomes when using immune cells. So, the determination of the endotoxin content of nanomaterials/nanomedicines before testing is essential (Giannakou et al. [39]).

Endotoxin or lipopolysaccharide (LPS) levels are restricted for parentally applied medicines and thus also for nanomedicines. Besides its direct toxic effect LPS is also an indicator for a possible bacterial contamination of the production process. This can be especially critical for nanomaterials as production may not always be performed under sterile conditions. However, for nanomedicines that in vivo are primarily taken up by phagocytizing cells like macrophages, the potential effect of LPS is even more critical. LPS is known for its ability to activate the immune system via its binding to the pathogen-pattern recognition receptor (PRR) toll-like receptor 4 (TLR4), the IL-1 receptor, as well as surface molecules like CD14 (Liebers et al. [40]; Zhang et al. [41]). For the detection of LPS, several biological assays are available. A classical standard test is the rabbit pyrogen test, which now has been replaced by an in vitro test, the most commonly used the limulus amebocyte lysate (LAL) test. Using the blood of the horseshoe crab, the LAL test is still dependent on animals as reagent source. More recently a chemical assay was developed based on measuring endotoxin indirectly via 3-hydroxylated fatty acids of lipid-A being the active part of the LPS, using ultrahigh performance liquid chromatography (UHPLC) coupled with mass spectrometry of specific phospholipids as part of the LPS molecule (Giannakou et al. [42]).

## The future

Above we have indicated a bright future for the development of new innovative nanomedicines both for specifically targeted disease (e.g., cancer) therapy and disease intervention by manipulating the immune system. The success of mRNA vaccines against COVID-19 was a game changer and only made possible by nanotechnology, described as “mRNA’s unsung partner” (May [43]; Vargason et al. [44]). In order to capitalize on the great opportunities, it should be realized that the introduction of new nanomedicinal entities can be problematic with regard to the preclinical safety evaluation to be performed before clinical trials and subsequently market authorization can be obtained. The REFINE project (Refine Nanomed — Regulatory Science Framework (refine-nanomed.eu)) has addressed a number of the issues discussed above specifically for nanomedicines. The project has identified regulatory challenges and gaps in available testing methods for nanomedicines. It has examined these issues in detail by using two model nanomaterials as carriers for active pharmaceutical ingredients, one carrier having a biodegradable polymeric structure (polymer class poly(alkyl cyanoacrylate)) and one carrier based on a lipid structure (LipImage™ 815). With these two model nanomedicines, a range of assays has been evaluated. In addition, important work has been performed on the development of physiologically based pharmacokinetic (PBPK) models. Also, a decision support system has been developed to help design the best characterization and testing strategy for nanomedicines. The work carried out in the project as presented in this special issue of *Drug Delivery and Translational Research* will provide an important contribution to further establishing science-based regulatory requirements for the safety evaluation of nanomaterials/nanomedicines.
